# Distal outcomes of communal goal mismatch: a longitudinal study of underrepresented students in STEM

**DOI:** 10.3389/fpsyg.2025.1483396

**Published:** 2025-05-21

**Authors:** Ashley Bonilla, Wesley Schultz, Anna Woodcock, Paul R. Hernandez

**Affiliations:** ^1^Division of Behavioral and Organizational Sciences, Claremont Graduate University, Claremont, CA, United States; ^2^Department of Teaching, Learning, and Culture, Texas A&M, College Station, TX, United States

**Keywords:** communal goal mismatch, STEM, belonging, intentions, identity

## Abstract

Using the goal congruity framework, this longitudinal study investigated whether communal goal mismatch predicted distal outcomes of STEM intentions, STEM identity, and a sense of belonging among students pursuing degrees in STEM fields. We hypothesized that students who experienced a negative mismatch (i.e., perceived insufficient opportunities to fulfill communal goals) would show lower intentions to pursue a STEM career, would be less likely to express a STEM identity, and would be less likely to feel like they belonged in their STEM field 1 year later. Results showed that students reported lower intentions to pursue the STEM fields when their perceived communal goal affordances in STEM were insufficient. They were also less likely to have a strong STEM identity. However, a negative mismatch did not significantly predict a sense of belonging. Overall, the results highlight the role of goal affordances in students’ academic and career choices.

## 1 Introduction

While in college, students must make important decisions that will determine the course of their academic careers. Among these is choosing a major. However, this decision alone does not guarantee that the student will earn the degree. For example, Deana Crouser was once one of the few Latinas majoring in chemical engineering at the University of Washington. Unfortunately, she reported that she spent more time worrying that she was not capable and that she did not belong, instead of focusing on her work. She eventually switched out of the STEM major because she felt excluded (EAB, 2021). Deana’s case is not uncommon. A study using data from a diverse sample of 5,600 Black, LatinX, and White people found that one third of students leave their STEM majors. But there are large equity gaps among students who choose to leave, with about 37% of LatinX students switching out of their STEM majors before earning a degree as compared to 29% of White people (Reigle-Crumb et al., 2019).

Social psychological theory and research has established the importance of several factors that can enhance the success of minority students in their pursuit of STEM degrees. Among these is a strong STEM identity (Schultz et al., 2011; Woodcock et al., 2012). A STEM identity refers to whether a person sees themselves as a scientist, an engineer, or a computer scientist, depending on their field. If an individual fails to form an identity with their STEM field, they are less likely to persist and are ultimately at risk of leaving the domain (Merolla et al., 2012; Stets et al., 2017; Woodcock et al., 2012). To illustrate, Dou et al. (2019) found that the odds that a student would choose a STEM career in college increased by 85% for every one-point higher scored on a STEM identity scale.

Whether students develop a strong STEM identity and a sense of belonging may be due to perceptions of alignment between personal goals and goals afforded in STEM. According to the Goal Congruity Framework (Diekman et al., 2010), individuals belonging to ethnic minorities in the U.S. are more likely to endorse communal goals which are other-focused goals that include helping others, collaboration, and ultimately benefiting the collective (Bakan, 1966; Thoman et al., 2015). However, STEM fields tend to be perceived as affording more agentic, or self-serving goals such as power, status, and recognition (Diekman et al., 2011). For this reason, students from ethnic minorities may be more likely to experience a negative communal goal mismatch, in which they perceive insufficient opportunities to fulfill their communal goals through a STEM career (Bonilla et al., 2023). This mismatch may influence whether they identify with their STEM domain, experience a sense of belonging, and ultimately whether or not they persist in a STEM field.

### 1.1 Literature review

#### 1.1.1 Goal congruity theory and communal goal mismatch

Career persistence is often influenced by how well an individual perceives a field supports their personal values and goals. The Goal Congruity Theory (Diekman et al., 2010) suggests that individuals seek to enter and engage in roles they perceive are aligned with their personal aspirations, especially if those careers offer opportunities to fulfill their dominant goals, whether they be agentic (e.g., power, achievement) or communal (e.g., helping others, collaboration). However, STEM fields are perceived to lack communal opportunities, therefore, leading to misalignment for students who prioritize communal values.

While prior research has investigated goal endorsements (e.g., Diekman et al., 2011; Lewis et al., 2019; Thoman et al., 2015) and goal affordances (e.g., Diekman et al., 2010; Henderson et al., 2022), to our knowledge, these constructs have not been examined together to assess the degree of alignment or misalignment between students’ personal goals and their perceptions of STEM affordances. To address this gap, Bonilla et al. (2023) introduced the concept of communal goal mismatch, a difference score measuring this alignment (communal goal affordance—communal goal endorsement). It differentiates experiences of mismatch by categorizing them as negative mismatch or positive mismatch focusing explicitly on communal goals. A negative mismatch indicates that the individual perceives STEM affords fewer communal opportunities than desired. A positive mismatch indicates the individual perceives STEM provides more communal opportunities than desired.

Introducing communal goal mismatch provides a novel framework for examining how individuals evaluate fit based on perceptions of alignment between personal goals and field affordances. Unlike prior research that examines goal endorsements and goal affordances separately, communal goal mismatch quantifies this misalignment, allowing for a more nuanced understanding of how mismatch can decrease engagement and perhaps conflict an individual’s sense of identity and belonging in STEM.

#### 1.1.2 Communal goal mismatch outside STEM

While the concept of communal goal mismatch is relatively new, goal congruity research has been applied in other fields where communal and agentic goal orientations influence career interest. For instance, Folberg et al. (2023) investigated how goal congruity shapes entrepreneurial interest among men and women, showing the individuals with strong communal orientations perceive entrepreneurship as less aligned with their values, ultimately affecting their venture interest. While Goal Congruity research has examined career decision-making in other fields, the concept of communal goal mismatch as a specific, quantified measure remains understudied. The findings from these adjacent fields suggest that similar mechanisms of alignment between goals endorsed and perceived opportunities may operate across various domains.

#### 1.1.3 Communal goal mismatch and STEM identity

STEM identity shapes whether individuals see themselves within STEM and plays a critical role in persistence, as those who do not strongly identify are more likely to leave the field (Merolla et al., 2012; Stets et al., 2017). STEM identity develops through internal self-perception (seeing oneself as a STEM individual) and external validation (being recognized as such by others) (Vincent-Ruz and Schunn, 2018). However, research has not fully explored how perceptions of goal alignment influence STEM identity development over time.

The sociological model of symbolic interactionism (Stryker, 2008) provides insight into how communal goal mismatch may weaken STEM identity. This framework posits that identities form through meaningful social interactions external validation influences how individuals see themselves in different contexts (Carter and Fuller, 2016). Therefore, when individuals experience a communal goal mismatch within STEM, they may lack the validation needed to reinforce their STEM identity, ultimately leading to difficulty in identifying with their STEM roles (i.e., engineer, scientist, mathematician, computer scientist). When students do not perceive communal opportunities, they may not see the field as a reflection of their personal values. Over time, this mismatch may weaken STEM identity and make it less likely that the individual continue pursuing a STEM career.

#### 1.1.4 Sense of belonging in STEM

Sense of belonging is also a fundamental predictor of STEM persistence (Belanger et al., 2020). Baumeister and Leary (1995) define sense of belonging as the feeling an individual experiences when they perceive they are being cared for and appreciated within a group. Applied to STEM, students feel valued and accepted within their respective STEM field. However, research has found disparities in belonging across demographic groups. Women and ethnic minorities often report a lower sense of belonging in STEM compared to men and White students (Good et al., 2012; Xu and Lastrapes, 2022). The doubt that historically marginalized groups feel about their acceptance in institutions (i.e., belonging uncertainty) particularly impacts these individuals (Walton and Cohen, 2011). Rainey et al. (2018) found that students who remained in STEM reported higher levels of sense of belonging compared to those who switched out, highlighting its importance for persistence.

Students who highly endorse communal goals often report a lower sense of belonging in STEM (Lewis et al., 2019). Belanger et al. (2020) found that highlighting communal opportunities in STEM increased sense of belonging and recalling communal opportunities helped individuals recover from threats to their sense of belonging in STEM. Their research suggests that addressing the perceived communal goal mismatch could enhance sense of belonging in STEM, especially for underrepresented groups.

Initial findings from an exploratory study (Bonilla et al., 2023) showed that negative communal mismatch (i.e., insufficient communal opportunities) was associated with a lower sense of belonging and lower STEM intentions. These results suggest that students who experience a negative communal goal mismatch may be less likely to persist in STEM, especially if their values are not reflected in the field.

While the goal congruity research has explored STEM persistence, few studies have examined how communal goal mismatch shapes STEM identity and belonging over time. Given this gap, the current study extends prior research by (1) using communal goal mismatch as a quantified construct, (2) examining STEM identity and belonging longitudinally, testing how communal goal mismatch predicts identity and belonging over time, (3) and exploring whether communal goal mismatch may disproportionately affect certain groups. Using longitudinal data from a large dataset of students pursing STEM degrees, we tested novel hypotheses about the role of communal goal mismatch in predicting distal outcomes of STEM intentions, sense of belonging, and STEM identity:

*(H1)* Negative communal goal mismatch would predict STEM intentions. Specifically, students who experience a greater negative mismatch would show lower levels of STEM intentions 1 year later.

*(H2)* Experiencing a negative communal goal mismatch would predict STEM identity, such that students that experience higher levels of negative communal goal mismatch will be less likely to express a STEM identity 1 year later (i.e., scientist/engineer/mathematician/software engineer/computer scientist).

*(H3)* Negative communal goal mismatch would predict student sense of belonging in STEM, such that students experiencing higher levels of negative communal goal mismatch would experience lower levels of sense of belonging 1 year later.

The hypotheses and data analytic plan were preregistered at: https://osf.io/wv8fy/?view_only=2af34421b7244a2e953b79d1fbcf0e50.

We also had three exploratory research questions: (1) Does race/ethnicity significantly moderate the relationship between communal goal mismatch and STEM intentions? (2) Does race/ethnicity significantly interact with communal goal mismatch to predict a STEM identity? (3) Does race/ethnicity significantly interact with communal goal mismatch to predict a STEM sense of belonging?

## 2 Methods

### 2.1 Participants

Participants were 1,310 undergraduate STEM students from 12 California State Universities from an ongoing 5-year longitudinal study, My College Pathways. The longitudinal panel was designed to examine differences in educational outcomes for White and LatinX people majoring in STEM, and predictors of career choice. Students in the panel were recruited during their junior year via email to all STEM majors from each university. Fifty-five percent reported being LatinX (45% White participants) and 51% were female (48% male, 1% other). The analytic sample (*n* = 838) was from the second and third year of the study (i.e., 2021–2022). Of the overall sample, *n* = 337 were omitted because they were categorized as “non-responders” for one or both surveys, *n* = 46 were omitted because they did not respond to the STEM identity measure, *n* = 60 were omitted because they did not respond to the STEM sense of belonging measure, *n* = 29 were omitted because they did not respond to the STEM intentions measure. In the third year, 17% reported having graduated with a bachelor’s degree and 83% were still enrolled. Approximately 44% were biological and life sciences majors, followed by engineering (37%), computer science (12.5%), math (5%), and 1.5% indicated having switched to a non-STEM major.

### 2.2 Power analysis

In an initial exploratory Bonilla et al. (2023), we found a small-to-medium sized relationship between negative mismatch and STEM intentions (*r* = 0.24) and no significant relationship for positive mismatch (*r* = 0.04). Drawing on our initial findings, we hypothesized a small association for H1 (STEM intentions) of standardized beta~0.10. Because our study utilized an existing longitudinal panel, we conducted a sensitivity analysis, showing that to achieve a power of 0.80, with a small effect size (beta = 0.10) would require a sample size of 617 (Cohen, 1992). Our analytic sample of 838 was sufficient to detect our hypothesized effect.

### 2.3 Procedure

Data for the current study were collected as part of wave-3 and wave-5 of the My College Pathways longitudinal study. Data were collected each semester. During recruitment, each student received an email invitation with a video that described the study. They then completed a screening survey (e.g., provided demographic information, STEM intentions) via an online Qualtrics survey (wave-0). To be eligible for the study, students had to be pursuing a STEM major at one of the 12 universities, identify as LatinX or White people, and had to be in their junior or senior year of college. Each student received $5 for their completion. Students who met the eligibility requirements were contacted the following spring (wave-1) and completed a longer survey (20-min).

Participants were surveyed during their spring 2021 (wave-3) and spring 2022 semesters (wave-5), receiving $20 for each survey completion. Of the 1,310 panel members, 838 participants (64%) completed all measures used for the current study at wave-3 and wave-5. They completed measures asking for additional demographic information, their communal goal endorsements, perceived communal goal affordances, STEM intentions, STEM identity, and their sense of belonging in their major (*science, engineering, software engineering, computer science, mathematics*).

### 2.4 Measures

All continuous predictor variables were centered prior to any analyses. Mean composite scores were also created for each outcome. For example, a mean STEM intentions score was created by averaging the scores of each of the items within the measure. This same procedure was used for communal goal endorsements and perceived communal goal affordance.

#### 2.4.1 Communal goal endorsement

Communal goal endorsements were measured with 7 communal items in the 23-item goal endorsement scale (Diekman et al., 2010): helping others, serving humanity, serving community, working with people, caring for others, connections with others, attending to others. Scale anchors ranged from 1 (*not at all important*) to 7 (*extremely important*). Reliability for the measure in wave-3 was 0.87.

#### 2.4.2 Agentic goal endorsement

Agentic goal endorsements were measured with 14 items in the 23-item goal endorsement scale (Diekman et al., 2010). Sample items include: power, recognition, achievement, and independence. Scale anchors ranged from 1 (not at all important) to 7 (extremely important). Reliability for the measure in wave-3 was 0.89.

#### 2.4.3 Perceived communal goal affordance

Perceived communal goal affordances were measured using 7 communal items in the 23-item goal affordance scale (Diekman et al., 2010): helping others, serving humanity, serving community, working with people, caring for others, connections with others, attending to others. Scale anchors ranged from 1 (*not at all*) to 7 (*extremely*). Reliability for the measure in wave-3 was 0.89.

#### 2.4.4 Perceived agentic goal affordance

Perceived agentic goal affordances were measured using 14 agentic items in the 23-item goal affordance scale (Diekman et al., 2010). Sample items include: power, recognition, achievement, and independence. Scale anchors ranged from 1 (not at all) to 7 (extremely). Reliability for the measure in wave-3 was 0.90.

#### 2.4.5 Communal goal mismatch

A communal goal mismatch score was calculated for each participant by subtracting their grand mean communal goal endorsement score from their grand mean perceived communal goal affordances. These scores ranged from-6 to 6 giving us two types of mismatch. A negative mismatch suggests that students perceived insufficient of opportunities to fulfill their personal communal goals (e.g., help others, work with others, attend to others). A positive mismatch indicates that students perceived a career in their major will afford them more opportunities than needed to fulfill their communal goals (e.g., help others, attend to others, connect with others). Communal goal mismatch type was dichotomized (0 = Negative, 1 = Positive). Note that the dichotomization of communal goal mismatch was only to code for the type of mismatch (either positive or negative, which we used as a moderator), and the full continuous score was used in our analyses.

#### 2.4.6 Agentic goal mismatch

An agentic goal mismatch score was calculated for each participant by subtracting their grand mean agentic goal endorsement score from their grand mean perceived agentic goal affordances. These scores ranged from-6 to 6 giving us two types of mismatch. A negative mismatch suggests that students perceived insufficient of opportunities to fulfill their personal agentic goals (e.g., gain recognition, money, power). A positive mismatch indicates that students perceived a career in their major will afford them more opportunities than needed to fulfill their communal goals (e.g., e.g., gain recognition, money, power).

#### 2.4.7 STEM intentions

STEM Intentions were measured using a 6-item scale (Schultz et al., 2011) that asks about future career plans. Sample items include: “To what extent do you intend to pursue a STEM-related research career?” Responses ranged from 0 (Definitely will not) to 10 (Definitely will) scale. Reliability for the measure in wave-5 was 0.83.

#### 2.4.8 STEM identity

STEM Identity was measured using Chemers et al. (2011) scale. Participants were asked to rate how they think about themselves and their identity as a [scientist/engineer/computer scientist/mathematician]. Sample items include: “In general, being [a scientist/an engineer/in a math related career/a computer scientist/a software engineer] is an important part of my self-image.” Scale anchors ranged from 1 (Strongly disagree) to a 5 (Strongly agree). Reliability for the measure in wave-5 was 0.90. The mean composite score for STEM identity was calculated by aggregating across engineering, computer science, science, mathematics, and software engineering identity scores. This same procedure was used to create the mean composite score for a STEM sense of belonging.

#### 2.4.9 Sense of belonging

Participants’ sense of belonging was measured using two-items from the Chemers et al. (2011) scale. Participants were asked to rate their agreement with two statements: “I feel like I belong in the field of [*science/ engineering /math/computer science /software engineering*]” and “I have a strong sense of belonging to the community of [*scientists/engineers/mathematicians/software engineers*].” Reliability for the measure in wave-5 was 0.81.

#### 2.4.10 Graduation status

Participants self-reported their graduation status. Prior to asking for graduation status, they were asked to confirm their enrollment status. If they selected “no longer enrolled,” they were then asked “Why are you not enrolled in a college or university right now?” Participants had the option of selecting “I graduated” therefore confirming their graduation status.

## 3 Results

### 3.1 Preliminary analyses

Prior to conducting any analyses, data were examined for normality, outliers, and multicollinearity. To investigate data normality, we used Malahanobis distances and compared the data to the critical values of the χ^2^ distribution. There were no values that surpassed the critical values, indicating that there were no issues of normality or any outliers. Our final analytical sample consisted of 838 participants.

Given that the data in our study were collected from 12 different campuses, a multilevel model was used to investigate whether the key variables of STEM intentions, identity, and belonging were clustered by campus. That is, whether the scores on each campus were more similar to each other than scores in general. This clustering effect can be quantified as an Intraclass Correlation Coefficient (ICC) and a high degree of clustering can produce an inflated probability of a Type-1 error. For STEM intentions, results from the intercept-only model showed that the average STEM intention score was 5.70 on a 0–10 scale. The intraclass correlation coefficient (ICC = 0.12) showed modest degree of clustering within the different campuses. Another intercept-only multilevel model was used to investigate whether STEM identity varied significantly across colleges. Results indicated that the average STEM identity score was 3.69 on a 5-point scale. The intraclass correlation coefficient (ICC = 0.002) indicated a negligible degree of clustering. Finally, a third multilevel model was used to investigate whether STEM sense of belonging varied significantly by campus. Results showed that the average STEM sense of belonging score was 3.67 on a 5-point scale. The intraclass correlation coefficient (ICC = 0.002) indicated a negligible degree of clustering. Taken together, the results do not suggest a high degree of clustering by campus, and we proceeded with our pre-registered analyses using hierarchical linear regression.

### 3.2 Main analyses

**Figure 1:**
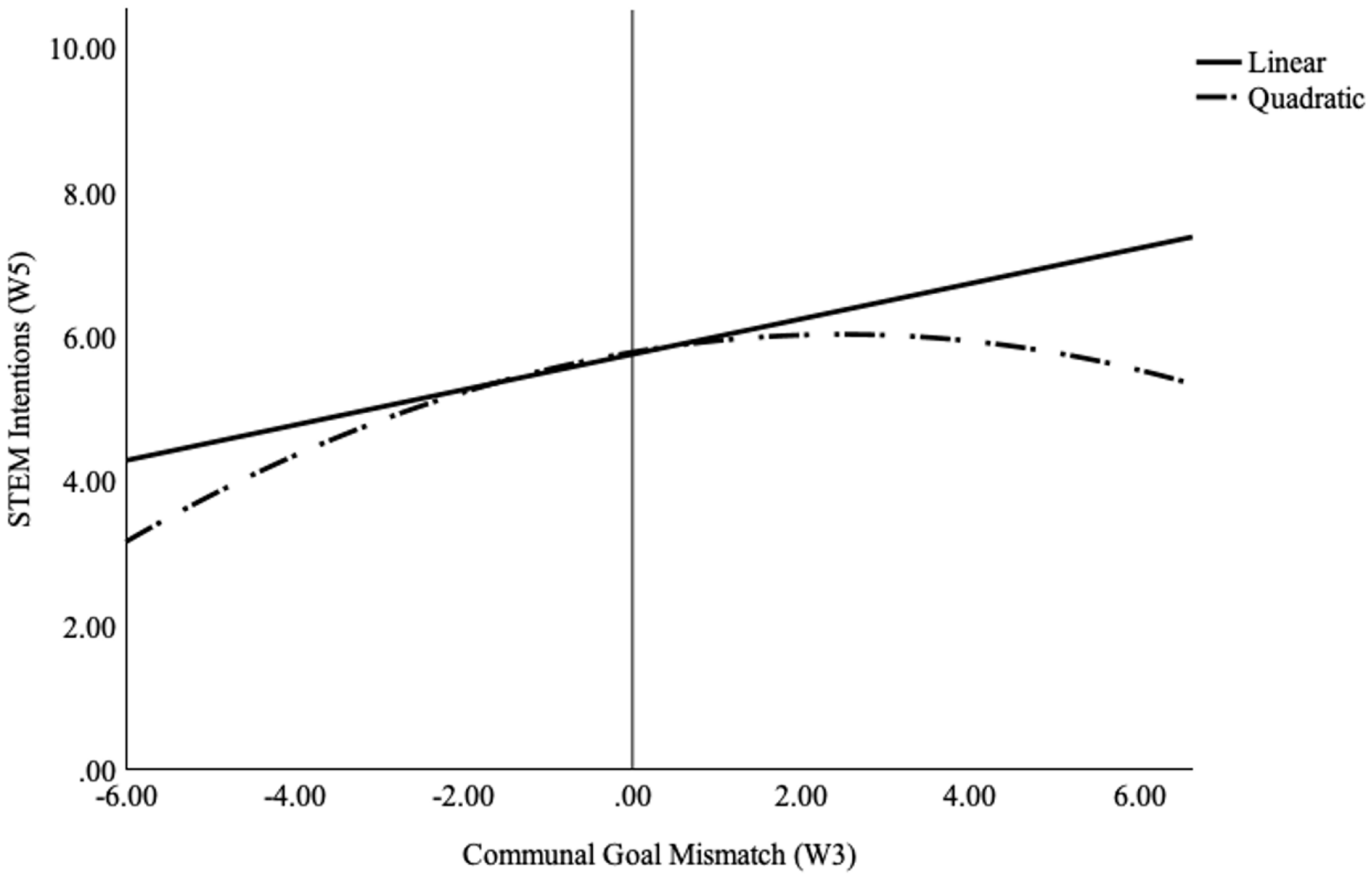
Communal goal mismatch score x mismatch type interaction on STEM intentions. Note: STEM Intentions is on a 0–10 scale. Communal goal mismatch scores range from-6 to 6. Negative mismatch suggests insufficient communal opportunities are perceived. Positive mismatch suggests an excess number of communal opportunities are perceived. Communal Goal Mismatch Score (W3) = Predictor from spring 2021 (wave-3). Mismatch Type (W3) = Predictor from spring 2021 (wave-3). STEM Intentions (W5) = intentions reported in spring 2022 (wave-5). * indicates *p* < 0.05.

Consistent with our pre-registration, a series of moderated regression analyses were conducted to test our hypotheses. Descriptive statistics and correlational analyses can be found in Table 1. Our first hypothesis predicted that students who experienced a greater negative mismatch would show lower levels of STEM intentions. To test this hypothesis, STEM intentions (wave-5) were entered as the dependent variable in the moderated regression model. Communal goal mismatch scores (wave-3) and communal goal mismatch type (wave-3; 0 = Negative, 1 = Positive) were entered in the first step of the regression. The communal goal mismatch score and mismatch type interaction term was entered in the second step of the regression. The overall model was significant, *F* (3, 834) = 5.29, *p* = 0.001. Type of communal goal mismatch (b = 0.60, 95% CI [−0.08, 1.13], SE = 0.27, beta = 0.13, *p* = 0.025) was a significant predictor of STEM intentions, indicating that whether a student experiences a positive (i.e., STEM can afford me more opportunities to fulfill communal goals than needed) or a negative communal goal mismatch (i.e., STEM does not afford me enough opportunities to fulfill communal goals) may influence their intentions. As hypothesized, the interaction of communal goal mismatch score and type of mismatch was statistically significant (b = −0.64; beta = −0.09, *p* < 0.05; see Supplementary Table 1). The pattern of the interaction was in the hypothesized direction: more negative mismatch was associated with lower levels of STEM intentions the following year. See Figure 1.

**Table 1:** Means, standard deviations, and correlations (*n* = 838).

Variable	*M*	*SD*	1	2	3	4	5	6	7	8	9	10	11	12
1. STEM ID (W3)	3.71	0.83	-											
2. Sense of Belonging (W3)	3.69	0.98	0.79**	-										
3. STEM Intentions (W3)	6.07	2.26	0.45**	0.34**	-									
4. Communal Goal Endorsement(W3)	5.22	0.88	0.38**	0.30**	0.30**	-								
5. Communal Goal Affordance (W3)	4.98	1.19	0.40**	0.35**	0.38**	0.59**	-							
6. Communal Goal Mismatch (W3)	−0.24	0.98	0.15**	0.15**	0.19**	−0.18**	0.69**	-						
7. STEM ID (W5)	3.69	0.91	0.70**	0.59**	0.37**	0.30**	0.31**	0.11**	-					
8. Sense of Belonging (W5)	3.67	1.03	0.61**	0.64**	0.29**	0.29**	0.31**	0.11**	0.83**	-				
9. STEM Intentions (W5)	5.70	2.37	0.39**	0.32**	0.68**	0.24**	0.26**	0.10**	0.50**	0.39**	-			
10. Communal Goal Endorsement (W5)	5.29	1.07	0.21**	0.18**	0.18**	0.55**	0.49**	0.09**	0.25**	0.25**	0.23**	-		
11. Communal Goal Affordance (W5)	4.82	1.21	0.35**	0.32**	0.29**	0.48**	0.67**	0.38**	0.4**	0.42**	0.35**	0.62**	-	

*M* and *SD* are used to represent mean and standard deviation, respectively. * indicates *p* < 0.05. ** indicates *p* < 0.01. Sense of belonging and STEM identity measures are on a 5 point scale. Perceived communal goal affordance and goal endorsement measures are on a 7-point scale. The STEM intentions measure is on a 10 point scale. Communal goal mismatch scores range from-6 to 6.

To illustrate the pattern of results, Figure 1 below shows the findings from a regression analysis including linear and quadratic terms from the full range of communal goal mismatch scores. The results showed that for communal mismatch scores that were negative (below the midpoint of zero), greater mismatch was associated with lower levels of STEM intentions. Similarly, for mismatch scores that were positive (meaning perceptions that STEM afforded more communal opportunities than the person desired), more mismatch was associated with lower levels of STEM intentions.

To test the second hypothesis that students who experience higher levels of negative communal goal mismatch would be less likely to express a STEM identity, an additional moderated regression was conducted. STEM identity was entered as the dependent variable. Communal goal mismatch scores and communal goal mismatch type were entered in the first step of the regression. The communal goal mismatch type and mismatch score interaction was entered in the second step. Results showed that the overall model was significant, F (3, 834) = 5.03, *p* = 0.002. Consistent with our hypothesis, the 2-way interaction between communal goal mismatch score and type of communal goal mismatch was a significant predictor of STEM identity (b = −0.24, 95% CI [−0.45, −0.03], SE = 0.11; beta = −0.09; *p* = 0.028; see Supplementary Table S2). The full results from the moderated regression are available in Supplementary Table S2.

**Figure 2:**
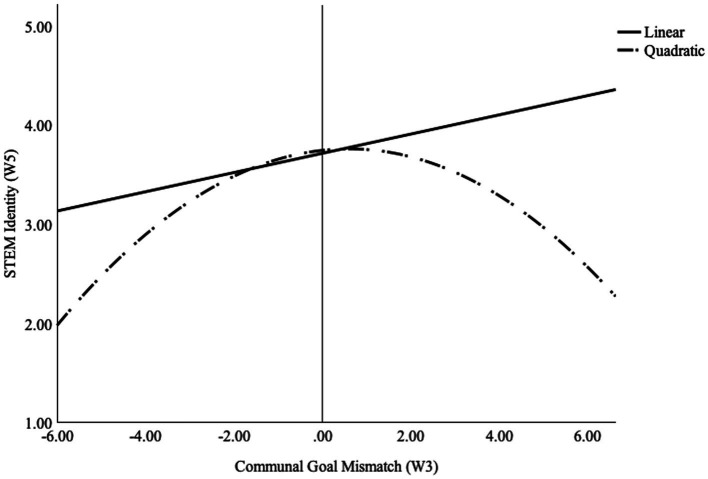
Communal goal mismatch score x mismatch type interaction on STEM identity. Note: STEM Identity is on a 1–5 scale. Communal goal mismatch scores range from-6 to 6. Negative mismatch suggests insufficient communal opportunities are perceived. Positive mismatch suggests an excess number of communal opportunities are perceived. Communal Goal Mismatch Score (W3) = Predictor from spring 2021 (wave-3). Mismatch Type (W3) = Predictor from spring 2021 (wave-3). STEM Identity (W5) = identity in spring 2022 (wave-5).

To illustrate the pattern of results, Figure 2 below shows the findings from a regression analysis including linear and quadratic terms from the full range of communal goal mismatch scores. The results showed that for communal mismatch scores that were negative (below the midpoint of zero), greater mismatch was associated with lower levels of STEM identity. Similarly, for mismatch scores that were positive (meaning perceptions that STEM afforded more communal opportunities than the person desired), more mismatch was associated with lower levels of STEM identity.

For the third moderated regression, the dependent variable was sense of belonging in STEM. Similar to the first two regression analyses, communal goal mismatch scores and mismatch type (from wave-3) were entered in the first step, and the interaction was entered in the second step. Results did not support our hypothesis. The overall model was significant, *F* (3, 834) = 4.71, *p* = 0.003, however, there were no significant predictors (see Supplementary Table S3).

Our final set of analyses explored the interaction of communal goal mismatch type and communal goal mismatch scores with race/ethnicity. Results showed no significant interaction of race/ethnicity, communal goal mismatch type, and communal goal mismatch score for STEM intentions (b = 1.08, 95% CI [−0.04, 2.20], SE = 0.57), STEM identity (b = 0.13, 95% CI [−0.31, 0.56], SE = 0.22), or STEM sense of belonging (b = −0.11, 95% CI [−0.60, 0.37], SE = 0.25). The full set of results are shown in Supplementary Table S4 (STEM intentions), Supplementary Table S5 (STEM identity), and Supplementary Table S6 (sense of belonging in STEM).

### 3.3 Exploratory analyses

We were interested in exploring whether communal goal mismatch may be related to graduation status. The analytical sample for these analyses was 839 participants. We computed a logistic regression analysis and all variables were grand-mean-centered. For this analysis, our analytical sample consisted of 115 participants because only those who indicated they were no longer enrolled were asked about their graduation status. Graduation status (0 = Not graduated, 1 = Graduated) at wave-5 (Spring 2022) was entered as the dependent variable. Centered communal goal mismatch scores (wave-3; Spring 2021), communal goal mismatch type (0 = Negative, 1 = Positive; wave-3 Spring 2021), and race/ethnicity (White or LatinX people) were entered into the model. In the second step, the communal goal mismatch score and communal goal mismatch type interaction, and the ethnicity and communal goal mismatch score interaction were entered. The 3-way interaction between ethnicity, communal goal mismatch type, and communal goal mismatch were entered in step three. The overall model was not significant, χ^2^(6) = 7.22, *p* = 0.301 and had an overall classification success rate of 90%. There were no significant predictors (see Supplementary Table S7).

For completeness of mismatch, we also tested the relationship between agentic goal mismatch at wave-3 (Spring 2021) on STEM identity at wave-5 (Spring 2022), STEM intentions at wave-5 (Spring 2022), and sense of belonging at wave-5 (Spring 2022). For these analyses, our analytical sample consisted of 838 participants. Results revealed that the same pattern emerged for all three outcomes. For STEM identity, our regression model with both linear and quadratic regression terms was statistically significant (*F* (2, 836) = 12.13, *p* < 0.001). The linear term was not significant, b = 0.04, SE(b) = 0.044, t = 0.88, *p* = 0.382. Results supported a curvilinear relationship, b = −0.15, SE(b) = 0.03, t = −4.74, p < 0.001, suggesting that the strength of the relationship between agentic goal mismatch and STEM identity varies for individuals who experience different types of mismatch. A positive mismatch would suggest that an individual perceives that STEM provides excess amounts of opportunities to fulfill agentic goals. A negative mismatch would suggest that an individual perceives STEM does not provide enough opportunities to fulfill agentic goals.

For STEM intentions at wave-5 (Spring 2022) our regression model with both linear and quadratic regression terms was also significant, F (2, 836) = 3.39, *p* = 0.034. The linear term was not significant, b = −0.09, SE(b) = 0.12, t = −0.78, *p* = 0.435. However, results supported a curvilinear relationship, b = −0.21, SE(b) = 0.08, t = −2.55, *p* = 0.011, suggesting that the strength of the relationship between agentic goal mismatch and STEM intentions varies for those who experience a positive or a negative mismatch.

Furthermore, when examining the relationship between agentic goal mismatch at wave-3 (Spring 2021) and sense of belonging (Spring 2022), a regression model with both linear and quadratic regression terms was significant [*F* (2, 836) = 9.06, *p* < 0.001]. The linear term was not significant (b = 0.05, SE(b) = 0.05, t = 1.06, *p* = 0.288), suggesting no direct linear relationship. However, the model supported a curvilinear relationship, b = −0.139, SE(b) = 0.04, t = −4.00, *p* < 0.001. Once again suggesting that the strength of the relationship between agentic goal mismatch and sense of belonging vary depending on the type of mismatch an individual experiences.

## 4 Discussion

The current study used a longitudinal dataset to investigate whether communal goal mismatch predicted distal outcomes of intentions to pursue STEM as a career field, a STEM identity, and a sense of belonging in STEM. Consistent with our preregistered hypothesis, when students experienced higher levels of negative communal goal mismatch, they reported lower intentions of pursuing the STEM fields 1 year later. This finding is consistent with a previous exploratory study that found that perceiving insufficient opportunities to fulfill communal goals (i.e., negative communal goal mismatch) was associated with lower STEM intentions (Bonilla et al., 2023), and with other research that has found that perceiving communal opportunities in STEM influences how students feel about STEM and their interest (Brown et al., 2018; Norman et al., 2022).

Our results also showed that when students experienced more negative mismatch, they were less likely to have a strong STEM identity (i.e., identify as a scientist, engineer, computer scientist, depending on their STEM field) 1 year later. This finding is both instructive and problematic, as previous research has shown the importance of STEM identity in predicting persistence and academic success (Dou et al., 2019; Merolla et al., 2012; Stets et al., 2017; Woodcock et al., 2012). If these students are struggling to identify with their STEM domain, they may be at risk of leaving the STEM fields altogether. These results highlight the role of goal perceptions and experiences of mismatch in shaping the academic and career trajectories of STEM students.

Although the interaction between type of communal goal mismatch and degree of mismatch were predictive of students STEM intentions and identities, they were not predictive of students’ sense of belonging in the STEM fields. Past exploratory research found that when students experienced a negative mismatch, they were less likely to feel like they belonged (Bonilla et al., 2023). Other work has shown the importance of a strong sense of belonging in STEM to persist and how a sense of belonging is higher among those who remain in STEM (Rainey et al., 2018). However, we found no significant relationships.

This null finding is noteworthy, suggesting that the psychological pathway from communal goal mismatch to STEM sense of belonging may not be as simple as with STEM identity. Students experiencing this communal goal mismatch may actively seek out supportive environments that align with their communal values within or outside of STEM. Perez et al. (2024) found that Latina STEM students who experienced incongruency between their personal communal goals and STEM’s individualistic nature, sought out environments to build connections with like-minded individuals. In some instances, students also reframed STEM affordances by identifying ways to integrate their communal values into their academic or professional goals. Rather than experiencing a decreased sense of belonging, some students adapted by creating spaces that support their values, buffering against the effects of inconsistencies.

Additionally, this finding may be related to the institutional context of our sample. Of the 23 university campuses within the California State University (CSU) system, twenty-one are Hispanic-Serving institutions and approximately 40% of the college student population within the CSU are Hispanic/LatinX (California State University’s Hispanic-serving Institutions; California State University, 2017). Research has shown that when a university celebrates racial/ethnic diversity, students may perceive they are welcomed in that environment and ultimately feel comfortable (Shaheed and Kiang, 2021).

Further, despite research showing differences of goal endorsements among ethnic groups (Thoman et al., 2015), we found no support for our exploratory analyses of interactions between race/ethnicity, type of communal goal mismatch, and communal goal mismatch score on STEM intentions, STEM identity, and a sense of belonging in STEM. However, these findings are consistent with a prior study that did not find significant differences in the relationship between communal goal mismatch and STEM intentions and the communal goal mismatch-sense of belonging relationship among White and LatinX people pursuing STEM (Bonilla et al., 2023). This may be due to the nature of the sample used in both studies (California State University students) as previously mentioned. Our results also failed to support our exploratory analyses of the relationship between communal goal mismatch scores, communal goal mismatch type, and graduation. A possible explanation could be that a majority of our sample have not yet graduated. However, this relationship may be important to explore in the future with a bigger sample size.

The additional exploratory analyses that we conducted between agentic goal mismatch and (1) STEM identity, (2) STEM intentions, and (3) sense of belonging all revealed that the degree of agentic goal mismatch an individual experiences influences their STEM identity, STEM intentions, and their sense of belonging. It is interesting to note that unlike with communal goal mismatch, there was a significant relationship between agentic goal mismatch and sense of belonging, suggesting the importance of alignment with agentic goals offered in STEM. Perhaps this is because STEM fields are stereotyped as agentic (Diekman et al., 2010). However, further exploration may be needed.

### 4.1 Limitations

Despite the clear findings of the current study, there are some limitations. First, all participants came from the CSU system that is recognized for being the most ethnically, economically, and academically diverse university system in the country (California State University, 2022). Almost 60% of the staff working at the CSU are LatinX and Black employees and more than half of the bachelor’s degrees the CSU awards are earned by LatinX, African American, or Native American students. Therefore, the universities within the system may not be representative of STEM departments at universities outside of the CSU. Additionally, our sample may not be representative of the college student population. Future research is warranted and should consider examining the communal goal mismatch experiences of students attending less ethnically diverse universities and include other populations like Black, Asian, and Indigenous students. Additionally, this study did not examine other potential contextual factors, such as faculty mentorship and institutional climate, which may influence how students perceive and navigate communal goal mismatch. Future research should examine how these factors interact with perceptions of communal goal mismatch.

A second limitation of the reported work is the focus on LatinX and White people majoring in STEM. The goal congruity literature has demonstrated that people perceive the STEM fields to be less communal and more agentic or self-serving (Diekman et al., 2010). It has also established that men and women have differing levels of goal endorsements, with women endorsing communal goals more highly (Diekman et al., 2010), which in turn tend to be negatively related to STEM interest. Given that the current study did not consider gender, we suggest future studies investigate the communal goal mismatch experiences of intersectional identities, such as women of color who are confronted with stereotypes that they are not as successful as men in STEM (Pietri et al., 2019) and are perceived as less competent than Asian and White people (McGee and Martin, 2011). These individuals are considered invisible, making it harder for them to envision their success in the STEM fields (Morton and Smith-Mutegi, 2018).

Another limitation of the current study was that we did not control for prior STEM intentions or STEM identity at wave-3 (Spring 2021), nor did we control for communal goal mismatch at wave-5 (Spring 2022). This was not initially considered in the pre-registered data analysis plan. Therefore, we avoided making any changes. We suggest future studies consider controlling for variables such as these. Additionally, this study treated communal goal mismatch as a dichotomous variable (negative vs. positive). A continuum-based approach could better capture these complexities and avoid oversimplifying students’ nuanced perceptions of goal alignment in STEM. Qualitative methods could also provide deeper insights into how students interpret and navigate perceived alignments with STEM fields.

## 5 Conclusion

Rather than accepting that students may experience a goal mismatch, it is important to investigate what aspects this influences and how it influences student pursuits. This research could potentially inform strategies to mitigate the consequences associated with experiencing goal mismatch and help increase diversity in the STEM fields. For example, having faculty within STEM departments that demonstrate the communal opportunities available within the STEM fields could help offset perceptions that STEM only affords agentic opportunities (Allen et al., 2021).

However, institutional resistance to shifting the long-standing STEM culture may pose a significant challenge, as STEM disciplines have historically prioritized agentic over communal values (Diekman et al., 2010). This resistance may stem from institutional traditions or significant restructuring. Overcoming initial resistance will require coordinated efforts across multiple levels and therefore, encourage university administrators to reward faculty who successfully incorporate communal approaches in their teaching and research. Future research should explore specific barriers to change, including, faculty attitudes, resource limitations, departmental policies, and other structures that hinder these changes.

To be more inclusive of goals, STEM curricula should be redesigned by integrating community-based research projects as requirements, encouraging collaborative problem-solving in addition to individual technical skills, and highlight the impact of the work being done at a societal level. Additionally, incorporating real-world applications (i.e., ways in which the STEM fields help improve quality of life and benefit others) in lesson plans can benefit students (Boucher et al., 2017). And finally, encouraging STEM faculty to behave in more communal ways can also help foster a greater sense of belonging and interest in the STEM fields (Norman et al., 2022).

Other ways in which we may be able to reduce the communal goal mismatch that students experience include providing hands-on opportunities (e.g., research assistant positions) that encourage students to work collaboratively with others. For example, after completing their first year, Harvey Mudd College gave their computer science students research opportunities. As a result of this, and accompanied by changes to their curriculum and conference travel, their graduation rate for female students in computer science jumped from 12% to about 40% within 5 years (Alvarado et al., 2012). It may be important to consider doing something similar in other fields that are lacking representation and have low graduation rates. Finally, it may also be helpful to encourage STEM professionals to share why they pursued the STEM fields and what they find most fulfilling about their work (Boucher et al., 2017). Doing so could help change the stereotypes associated with the STEM fields and motivate students majoring in STEM to persist, ultimately increasing representation.

The persistent underrepresentation in STEM represents a significant loss of talent and perspective that limits innovation and scientific progress. We urge educators and administrators to commit to restructuring STEM to be more inclusive, creating pathways for all students to contribute their unique talents and perspectives.

## Data Availability

The datasets presented in this study can be found in online repositories. The names of the repository/repositories and accession number(s) can be found in the article/Supplementary material.
