# Supplementary material for: Distal outcomes of communal goal mismatch: a longitudinal study of underrepresented students in STEM
Source: Front Psychol. 2025 May 21;16:1483396. doi: 10.3389/fpsyg.2025.1483396 (PMC12135338; doi:10.3389/fpsyg.2025.1483396)
Supplement: Supplementary file 1 [file Supplementary_file_1.docx]

**Table S1**

*Moderation Regression Results for STEM Intentions*

| Variable | *b* | *95% CI for b* | | *SE b* | *ß* | *R^2^* | *ΔR^2^* |
| --- | --- | --- | --- | --- | --- | --- | --- |
|  |  | *LL* | *UL* |  |  |  |  |
| Model 1 |  |  |  |  |  | 0.01 |  |
| Constant | 5.70 | 5.54 | 5.86 | 0.08 |  |  |  |
| Mismatch Type  Mismatch Score | 0.35  0.11 | -0.13  -0.13 | 0.83  0.36 | 0.24  0.12 | 0.07 |  |  |
| Model 2 |  |  |  |  |  | 0.02 | 0.006^*^ |
| Constant | 5.93 | 5.67 | 6.19 | 0.13 |  |  |  |
| Mismatch Type | 0.60 | 0.08 | 1.13 | 0.27 | 0.13* |  |  |
| Mismatch Score  Mismatch Type x Score | -0.02  -0.64 | -0.29  -1.20 | 0.25  -0.08 | 0.14  0.28 | -0.01  -0.09* |  |  |

*Note.* Constant = intercept. Mismatch Type = type of communal goal mismatch (i.e., negative vs. positive). Mismatch Score = communal goal mismatch score. Mismatch Type x Score = communal goal mismatch type and communal goal mismatch score interaction. Centered data were used. *LL* and *UL* indicate the lower and upper limits of a confidence interval, respectively. * indicates *p* < .05. ** indicates *p* < .01. *** indicates *p* < .001.

**Table S2**

*Moderated Regression Results for STEM Identity*

| Variable | *b* | *95% CI for b* | | *SE b* | *ß* | *R^2^* | *ΔR^2^* |
| --- | --- | --- | --- | --- | --- | --- | --- |
|  |  | *LL* | *UL* |  |  |  |  |
| Model 1 |  |  |  |  |  | 0.01 |  |
| Constant | 3.69 | 3.63 | 3.76 | 0.03 |  |  |  |
| Mismatch Type  Mismatch Score | 0.08  0.06 | -0.02  -0.13 | 0.17  0.24 | 0.05  0.09 | 0.08  0.03 |  |  |
| Model 2 |  |  |  |  |  | 0.02 | 0.006^*^ |
| Constant | 3.78 | 3.68 | 3.88 | 0.05 |  |  |  |
| Mismatch Type | 0.25 | -0.08 | 0.13 | 0.05 | 0.03 |  |  |
| Mismatch Score  Mismatch Type x Score | 0.15  -0.24 | -0.05  -0.46 | 0.36  -0.03 | 0.10  0.11 | 0.08  -0.09* |  |  |

*Note.* Constant = intercept. Mismatch Type = type of communal goal mismatch (i.e., negative vs. positive). Mismatch Score = communal goal mismatch score. Mismatch Type x Score = communal goal mismatch type and communal goal mismatch score interaction. Centered data were used. *LL* and *UL* indicate the lower and upper limits of a confidence interval, respectively. * indicates *p* < .05. ** indicates *p* < .01. *** indicates *p* < .001.

**Table S3**

*Moderated Regression Results for STEM Sense of Belonging*

| Variable | *b* | *95% CI for b* | | *SE b* | *ß* | *R^2^* | *ΔR^2^* |
| --- | --- | --- | --- | --- | --- | --- | --- |
|  |  | *LL* | *UL* |  |  |  |  |
| Model 1 |  |  |  |  |  | 0.01 |  |
| Constant | 3.67 | 3.60 | 3.74 | 0.04 |  |  |  |
| Mismatch Type  Mismatch Score | -0.05  0.14 | -0.26  0.03 | 0.15  0.24 | 0.11  0.05 | -0.02  0.13 |  |  |
| Model 2 |  |  |  |  |  | 0.02 | 0.004 |
| Constant | 3.76 | 3.64 | 3.87 | 0.06 |  |  |  |
| Mismatch Type | 0.04 | -0.19 | 0.27 | 0.12 | 0.02 |  |  |
| Mismatch Score  Mismatch Type x Score | 0.09  -0.23 | -0.03  -0.48 | 0.20  0.01 | 0.06  0.12 | 0.08  -0.07 |  |  |

*Note.* Constant = intercept. Mismatch Type = type of communal goal mismatch (i.e., negative vs. positive). Mismatch Score = communal goal mismatch score. Mismatch Type x Score = communal goal mismatch type and communal goal mismatch score interaction. Centered data were used. *LL* and *UL* indicate the lower and upper limits of a confidence interval, respectively. * indicates *p* < .05. ** indicates *p* < .01. *** indicates *p* < .001.

**Table S4**

*Moderated Regression Results for STEM Intentions*

| Variable | *b* | *95% CI for b* | | *SE b* | *ß* | *R^2^* | *ΔR^2^* |
| --- | --- | --- | --- | --- | --- | --- | --- |
|  |  | *LL* | *UL* |  |  |  |  |
| Model 1 |  |  |  |  |  | 0.02 |  |
| Constant | 5.70 | 5.54 | 5.86 | 0.08 |  |  |  |
| Mismatch Score  Mismatch Type  Ethnicity | 0.10  0.34  -0.28 | -0.14  -0.14  -0.60 | 0.35  0.82  0.04 | 0.12  0.24  0.16 | 0.43  0.71  -0.06 |  |  |
| Model 2 |  |  |  |  |  | 0.02 | 0.007 |
| Constant | 5.93 | 5.67 | 6.18 | 0.13 |  |  |  |
| Mismatch Score | -0.04 | -0.31 | 0.23 | 0.14 | -0.02 |  |  |
| Mismatch Type  Ethnicity  Mismatch type x Score  Ethnicity x Mismatch Type  Ethnicity x Mismatch Score | 0.59  -0.27  -0.60  -0.42  0.29 | 0.06  -0.59  -1.16  -1.38  -0.20 | 1.12  0.05  -0.05  0.55  0.78 | 0.27  0.16  0.28  0.49  0.25 | 0.13*  -0.06  -0.08*  -0.04  0.06 |  |  |
| Model 3 |  |  |  |  |  | 0.03 | 0.004 |
| Constant  Mismatch Score  Mismatch Type  Ethnicity  Mismatch type x Score  Ethnicity x Mismatch Type  Ethnicity x Mismatch Score  Ethnicity x Type x Score | 5.93  -0.03  0.59  -0.66  -0.63  -0.85  0.52  1.08 | 5.67  -0.31  0.06  -1.18  -1.19  -1.91  -0.02  -0.04 | 6.19  0.24  1.12  -0.14  -0.74  0.21  1.06  2.20 | 0.13  0.14  0.27  0.26  0.28  0.54  0.28  0.57 | -0.14  0.13*  -0.14*  -0.09*  -0.09  0.11  0.11 |  |  |

*Note.* Constant = intercept. Mismatch Score = communal goal mismatch score. Mismatch Type = communal goal mismatch type (i.e., negative vs. positive). Ethnicity = Hispanic or White. Mismatch type x Score = Communal goal mismatch score and communal goal mismatch type interaction. Ethnicity x Mismatch Type = Ethnicity and communal goal mismatch type interaction. Ethnicity x Mismatch Score = Ethnicity and communal goal mismatch score interaction. Ethnicity x Type x Score = Ethnicity, communal goal mismatch type, and communal goal mismatch score 3-way interaction. Centered data were used. *LL* and *UL* indicate the lower and upper limits of a confidence interval, respectively. * indicates *p* < .05. ** indicates *p* < .01. *** indicates *p* < .001.

**Table S5**

*Moderated Regression Results for STEM Identity*

| Variable | *b* | *95% CI for b* | | *SE b* | *ß* | *R^2^* | *ΔR^2^* |
| --- | --- | --- | --- | --- | --- | --- | --- |
|  |  | *LL* | *UL* |  |  |  |  |
| Model 1 |  |  |  |  |  | 0.01 |  |
| Constant | 3.69 | 3.63 | 3.76 | 0.03 |  |  |  |
| Mismatch Score  Mismatch Type  Ethnicity | 0.08  0.05  -0.04 | -0.02  -0.13  -0.16 | 0.17  0.24  0.09 | 0.05  0.09  0.06 | 0.08  0.03  -0.02 |  |  |
| Model 2 |  |  |  |  |  | 0.02 | 0.007 |
| Constant | 3.78 | 3.68 | 3.89 | 0.05 |  |  |  |
| Mismatch Score | 0.03 | -0.08 | 0.13 | 0.05 | 0.03 |  |  |
| Mismatch Type  Ethnicity  Mismatch type x Score  Ethnicity x Mismatch Type  Ethnicity x Mismatch Score | 0.15  -0.03  -0.24  -0.17  0.04 | -0.06  -0.15  -0.46  -0.54  -0.15 | 0.35  0.10  -0.02  0.21  0.23 | 0.10  0.06  0.11  0.19  0.10 | 0.08  -0.02  -0.08*  -0.05  0.02 |  |  |
| Model 3 |  |  |  |  |  | 0.02 | 0.001 |
| Constant  Mismatch Score  Mismatch Type  Ethnicity  Mismatch type x Score  Ethnicity x Mismatch Type  Ethnicity x Mismatch Score  Ethnicity x Type x Score | 3.78  0.03  0.15  -0.08  -0.24  -0.22  0.07  0.13 | 3.68  -0.08  -0.06  -0.28  -0.46  -0.63  -0.15  -0.31 | 3.88  0.13  0.35  0.12  -0.03  0.19  0.27  0.56 | 0.05  0.05  0.10  0.10  0.11  0.21  0.11  0.22 | 0.03  0.08  -0.04  -0.09*  -0.06  0.04  0.03 |  |  |

*Note.* Constant = intercept. Mismatch Score = communal goal mismatch score. Mismatch Type = communal goal mismatch type (i.e., negative vs. positive). Ethnicity = Hispanic or White. Mismatch type x Score = Communal goal mismatch score and communal goal mismatch type interaction. Ethnicity x Mismatch Type = Ethnicity and communal goal mismatch type interaction. Ethnicity x Mismatch Score = Ethnicity and communal goal mismatch score interaction. Ethnicity x Type x Score = Ethnicity, communal goal mismatch type, and communal goal mismatch score 3-way interaction. Centered data were used. *LL* and *UL* indicate the lower and upper limits of a confidence interval, respectively. * indicates *p* < .05. ** indicates *p* < .01. *** indicates *p* < .001.

**Table S6**

*Moderated Regression Results for STEM* *Sense of Belonging*

| Variable | *b* | *95% CI for b* | | *SE b* | *ß* | *R^2^* | *ΔR^2^* |
| --- | --- | --- | --- | --- | --- | --- | --- |
|  |  | *LL* | *UL* |  |  |  |  |
| Model 1 |  |  |  |  |  | 0.01 |  |
| Constant | 3.67 | 3.60 | 3.74 | 0.04 |  |  |  |
| Mismatch Score  Mismatch Type  Ethnicity | 0.14  -0.05  0.01 | 0.03  -0.26  -0.13 | 0.24  0.16  0.15 | 0.05  0.11  0.07 | 0.13  -0.03  0.01 |  |  |
| Model 2 |  |  |  |  |  | 0.02 | 0.005 |
| Constant | 3.76 | 3.65 | 3.87 | 0.06 |  |  |  |
| Mismatch Score | 0.08 | -0.03 | 0.20 | 0.06 | 0.08 |  |  |
| Mismatch Type  Ethnicity  Mismatch type x Score  Ethnicity x Mismatch Type  Ethnicity x Mismatch Score | 0.04  0.02  -0.23  -0.01  0.04 | -0.19  -0.12  -0.47  -0.42  -0.17 | 0.27  0.16  0.01  0.41  0.26 | 0.12  0.07  0.12  0.21  0.11 | 0.02  0.01  -0.07  -0.01  0.02 |  |  |
| Model 3 |  |  |  |  |  | 0.02 | 0.001 |
| Constant  Mismatch Score  Mismatch Type  Ethnicity  Mismatch type x Score  Ethnicity x Mismatch Type  Ethnicity x Mismatch Score  Ethnicity x Type x Score | 3.76  0.08  0.04  0.06  -0.23  0.04  0.02  -0.11 | 3.64  -0.04  -0.19  -0.17  -0.47  -0.42  -0.22  -0.60 | 3.87  0.20  0.27  0.29  0.02  0.50  0.26  0.37 | 0.06  0.06  0.12  0.12  0.12  0.24  0.12  0.25 | 0.08  0.02  0.03  -0.07  0.01  0.01  -0.03 |  |  |

*Note.* Constant = intercept. Mismatch Score = communal goal mismatch score. Mismatch Type = communal goal mismatch type (i.e., negative vs. positive). Ethnicity = Hispanic or White. Mismatch type x Score = Communal goal mismatch score and communal goal mismatch type interaction. Ethnicity x Mismatch Type = Ethnicity and communal goal mismatch type interaction. Ethnicity x Mismatch Score = Ethnicity and communal goal mismatch score interaction. Ethnicity x Type x Score = Ethnicity, communal goal mismatch type, and communal goal mismatch score 3-way interaction. Centered data were used. *LL* and *UL* indicate the lower and upper limits of a confidence interval, respectively. * indicates *p* < .05. ** indicates *p* < .01. *** indicates *p* < .001.

**Table S7**

*Binary Logistic Regression Results for Graduation Status*

| Variable | *b* | *SE b* | *Wald χ^2^* | *p* | *Odds Ratio* |
| --- | --- | --- | --- | --- | --- |
|  |  |  |  |  |  |
| Model 1 |  |  |  |  |  |
| Constant | 2.22 | 0.58 | 14.58 | .001*** | 9.23 |
| Ethnicity | 1.46 | 1.32 | 1.23 | .268 | 4.31 |
| Mismatch Score  Mismatch Type  Mismatch Type x Score  Ethnicity x Mismatch Score  Ethnicity x Type x Score | -0.58  -0.37  2.47  -3.58  0.89 | 1.93  1.24  3.70  4.12  7.90 | 0.09  0.09  0.47  0.76  0.01 | .764  .768  .504  .384  .910 | 0.56  0.69  11.84  0.03  2.44 |

*Note.* Constant = intercept. Mismatch Score = communal goal mismatch score. Mismatch Type = communal goal mismatch type (i.e., negative vs. positive). Ethnicity = Hispanic or White. Mismatch type x Score = Communal goal mismatch score and communal goal mismatch type interaction. Ethnicity x Mismatch Type = Ethnicity and communal goal mismatch type interaction. Ethnicity x Mismatch Score = Ethnicity and communal goal mismatch score interaction. Ethnicity x Type x Score = Ethnicity, communal goal mismatch type, and communal goal mismatch score 3-way interaction. Centered data were used. * indicates *p* < .05. ** indicates *p* < .01. *** indicates *p* < .001.
